# The impact of low-pressure pneumoperitoneum on robotic-assisted radical cystectomy and intracorporeal ileal conduit urinary diversion: a case–control study

**DOI:** 10.1007/s00345-022-04117-w

**Published:** 2022-09-05

**Authors:** Nikolaos Kostakopoulos, Grigorios Athanasiadis, Muhammad Imran Omar, Jacalyn Abraham, Konstantinos Dimitropoulos

**Affiliations:** 1 Department of Urology, Aberdeen Royal Infirmary, NHS Grampian, Aberdeen, Scotland, UK; 2 Academic Urology Unit, University of Aberdeen, Aberdeen, Scotland, UK

**Keywords:** Low-pressure, Pneumoperitoneum, Intra-abdominal pressure, Robotic, Radical cystectomy, Intracorporeal urinary diversion

## Abstract

**Purpose:**

To evaluate the role of low intra-abdominal pressure (IAP) in improving postoperative recovery in Robotic-assisted radical cystectomy (RARC) and intracorporeal ileal conduit urinary diversion (ICUD).

**Methods:**

A retrospective case–control study of 49 bladder cancer patients offered RARC/ICUD with standard (12 mmHg, *n* = 24) or low IAP (8 mmHg, *n* = 25). Outcomes of interest included length of procedure (LoP), estimated blood loss (EBL), blood transfusion, margin positivity rates, time to first flatus (TtFF), time to first bowel movement (TtFBM), ileus and small bowel obstruction (SBO) rates, time to safe discharge (TtSD), postoperative hospital stay (PHS) and pain levels on a postoperative day (POD) 1 and 3. Perioperative complications were recorded using the Clavien-Dindo system.

**Results:**

Demographic and baseline clinical characteristics, LoP, EBL and margin positivity rates were similar between groups. No transfusions were recorded. Median (IQR) TtFF, TtFBM and TtSD were significantly longer in Group 1 vs Group 2 (4 (1) vs 2 (1), 7 (3) vs 6 (2) and 8.5 (5.75) vs 5.0 (1), respectively). PHS and rates of postoperative ileus and SBO were lower in Group 2, however not statistically significant. Severe pain was uncommon in both groups but moderate/severe pain was significantly higher in Group 1 (95.8% vs 48% on POD1 and 62.5% vs 16% on POD3). No significant intraoperative complications were recorded and ≥ Grade 3 postoperative complications at 30 and 90 days were similar.

**Conclusion:**

With limitations, Low-IAP RARC can be safely offered to RARC/ICUD patients and leads to faster bowel recovery, and shorter time to safe discharge compared to standard pneumoperitoneum.

## Introduction and objectives

Establishment of pneumoperitoneum represents the basis of all laparoscopic (conventional and robotic-assisted) procedures. It creates the necessary working space in the abdomen/pelvis, helps with smoke evacuation and delineation of the tissue planes, and reduces blood loss by compressing the bleeding vessels [1].

However, high intra-abdominal pressures can lead to elevation of the diaphragm and lung base collapse, problems with ventilation, compression of the Inferior Vena Cava and hypercarpnia/respiratory acidosis [2, 3]. Of importance, compression of the bowel loops (especially prolonged one) can lead to the development of postoperative ileus and tissue ischaemia [4]. Moreover, it is not uncommon for patients to report significant abdominal and shoulder tip pain postoperatively, due to the pneumoperitoneum-mediated overstretching of the abdominal and diaphragmatic muscle fibres [5].

The impact of low-pressure pneumoperitoneum on the outcomes of robotic urological procedures such as radical prostatectomy and upper urinary tract robotic surgery has been recently investigated [6, 7]. However, there is a lack of evidence regarding the role of low IAP in Robotic-Assisted Radical Cystectomy (RARC) and intracorporeal ileal conduit urinary diversion (ICUD). While low IAP could in theory eliminate some of the pneumoperitoneum-related side effects and complications (patient discomfort and pain, ileus), it remains unclear whether this could adversely affect the perioperative parameters (limited working space, higher rates of intra- and postoperative complications, higher blood loss).

In this single-centre retrospective study, we evaluated the role of low intra-abdominal pressure (IAP) in the improvement of postoperative RARC recovery.

## Patients and methods

This is a retrospective review of RARC/ICUDs performed by the same surgeon (KD) between January 2021 and February 2022 in our institution. The manuscript was drafted in line with the STROBE (Strengthening The Reporting of Observational Studies in Epidemiology) checklist for the case series [8].

Adult patients with a diagnosis of muscle- or non-muscle-invasive bladder cancer (MIBC and NMIBC, respectively) and indications for bladder removal with a formation of ileal conduit were included in this case–control study. If MIBC patients were deemed fit, Neo-Adjuvant Chemotherapy (3–4 cycles of Gemcitabine and Cisplatin) was offered to them. After retrospective review and collection of the data, patients were divided into 2 groups: Group 1 included patients who were offered RARC and ICUD with IAP at 12 mmHg, while Group 2 patients were offered an operation with IAP at 8 mmHg. It needs to be highlighted that low and standard IAP cases were mixed and allocation to Groups was retrospectively done based on the IAP that was selected by the surgeon in theatre. Patients who were offered additional procedures, such as synchronous radical nephroureterectomy, and patients who were offered palliative/simple cystectomies were excluded to keep the pool of patients as homogenous as possible.

Radical cystoprostatectomy/cystectomy (with or without total hysterectomy in women) and extended pelvic lymphadenectomy were performed with the use of the DaVinci Xi surgical system (Intuitive Surgical). In all patients, a surgeon-controlled bipolar vessel-sealing device (Da Vinci Vessel Sealer Extend or Synchroseal, Intuitive Surgical) was used to control the bladder (and in men, prostatic) pedicles. The dorsal venous complex in men was oversewn with a haemostatic suture prior to transection while in women the bipolar vessel-sealing device was used for safe dissection of the vessels.

Trendelenburg position on the surgical table was then reduced from 25° to 13–15° and 2 pairs of stay sutures were placed in the proximal and distal conduit end sites. Robotic instruments were used to hold the sutures and help with the manipulation of the ileal loops during ICUD, minimising direct grasping of the ileum and mesentery. Isolation of the 15-20 cm conduit loop and formation of the ileoileal anastomosis (Barcelona technique) was performed with the use of a laparoscopic powered stapling system (Signia Stapling System, Medtronic). A stable pneumoperitoneum insufflation and continuous smoke evacuation system was used in all cases (Airseal iFS, ConMed). IAP was set at 8 mmHg for the radical cystoprostatectomy/cystectomy and extended pelvic lymphadenectomy while ICUD was performed with an IAP of 6-8 mmHg. IAP would be raised to 15-18 mmHg > 2–5’ in case of a surgical emergency such as a significant bleeding event or following the surgeon’s request.

Enhanced Recovery After Surgery Protocol was followed postoperatively, in keeping with current practice [9]. Postoperative analgesia included a combination of paracetamol and oral opioids (usually oxycodone), regular or as needed, depending on postoperative anaesthetic instructions. Immediate mobilisation of the patients and the use of chewing gum was encouraged [10]. Patients would have a liquid diet on Postoperative Day (POD) 1, soft diet on PODs 2 and 3 and were re-established on a normal diet on POD4. Pelvic drain was removed on POD 2/3 and removal of stents was done on POD7-10.

Demographic and relevant clinical parameters such as age of the patient, BMI, TNM classification, use of NAC and American Society of Anesthesiology (ASA) scores were collected [11, 12]. Study outcomes of interest comprised of the length of procedure (LoP), estimated blood loss (EBL), blood transfusion and margin positivity rates, time to first flatus (TtFF), time to first bowel movement (TtFBM), postoperative ileus and small bowel obstruction (SBO) rates, time to safe discharge (TtSD, defined as the duration of hospital stay until patient is deemed medically stable for discharge) and total postoperative hospital stay (PHS). Postoperative pain levels were recorded on PODs 1 and 3 using a 3-point scale (mild, moderate, severe). Perioperative complications at 30 and 90 days were recorded using the Clavien-Dindo classification system [13].

Statistical analysis of the data was performed with the use of the SPSS statistical software and non-parametric tests were used to compare continuous and categorical variables (Mann–Whitney U and χ^2^ test, respectively), between the study groups.

## Results

In total, 55 consecutive patients who underwent RARC and ICUD by the same surgeon (KD) were identified. A total of 6 patients were excluded (4 synchronous nephroureterectomies, one palliative anterior exenteration/IUD and one case that required extracorporeal diversion due to subhepatic position of the caecum). Retrospective review of the remaining 49 electronic patient records showed 24 patients in Group 1 and 25 patients in Group 2. In total, 37/49 (75.51%) of patients had muscle-invasive bladder cancer; of them, 78.4% (29/37) were offered and completed neo-adjuvant chemotherapy preoperatively (13 and 16 in Group 1 and 2, respectively, *p* > 0.05).

Demographic parameters and baseline clinical characteristics were all similar between groups (*p* > 0.05, Table 1). In this cohort of patients, 1 patient had previous radical radiotherapy for bladder cancer (Group 2), 2 patients had radical radiotherapy for prostate cancer (1 in each group) and one Group 1 patient had a previous robotic-assisted radical prostatectomy. Two patients required early conversion from low to standard pneumoperitoneum as low IAP failed to control ongoing ooze from the pelvic tissue planes. These patients were handled as standard IAP cases.

**Table 1:** Demographic and baseline clinical characteristics

	Group 1 – Std IAP (*n* = 24)	Group 2 – Low IAP (*n* = 25)	*p*
Sex			
Male	17 (70.8%)	17 (68.0%)	0.999
Female	7 (29.2%)	8 (32.0%)	
Age (years)*	69.50 (21.25)	71.00, (14.00)	0.210
BMI (kg/m^2^)*	28.10 (8.85)	26.70 (7.40)	0.496
ASA score			
I	1 (4.2%)	1 (4.0%)	0.997
II	19 (79.2%)	20 (80.0%)	
III	4 (16.7%)	4 (16.0%)	
Preoperative Clinical Staging			
VHR/BCGu NMIBC	6 (25.0%)	6 (24.0%)	0.890
MIBC *(T2)*	13 (54.2%)	15 (60.0%)	
Locally Advanced *(T3-4, N1-3)*	5 (20.8%)	4 (16.0%)	
Patients who were offered and completed NAC	13 (54.2%)	16 (64.0%)	0.567

*IAP* intra-abdominal pressure, *BMI* body mass index, *ASA* American society of anesthesiology, *VHR* very-high risk, *BCGu* BCG unresponsive, *NMIBC* non-muscle invasive bladder cancer, *MIBC* muscle-invasive bladder cancer, *NAC* neo-adjuvant chemotherapy

*Median(InterQuartile Range)

Table 2 presents the results of the perioperative data analysis. Between-group comparisons showed significantly higher Median/IQR TtFF, TtFBM and TtSD (in days) in Group 1 vs Group 2 (4 (1) vs 2 (1), *p* = 0.001, 7 (3) vs 6 (2), *p* = 0.046, and 8.5 (5.75) vs 5.0 (1), *p* = 0.001, respectively), however, LoS was similar between groups (*p* > 0.05). Regarding bowel complications, rates of postoperative ileus and SBO were lower in Group 2, however, the difference did not reach statistical significance [16% vs 29.2% and 0% vs 8.3%, respectively, *p* > 0.05).

**Table 2:** Intra- and postoperative parameters

	Group 1 – Std IAP	Group 2 – Low IAP	*p*
Length of Procedure (min) ^*^	315 (93)	300 (90)	0.218
EBL (cc) ^*^	240 (325)	200 (600)	0.554
Time to First Flatus (days)	4 (1)	2 (1)	0.001
Time to First Bowel Movement (days)	7 (3)	6 (2)	0.046
Postoperative Ileus (*n*, %)	7 (29.2%)	4 (16%)	0.321
Postoperative SBO (*n*, %)	2 (8.33%)	0 (0%)	0.235
Positive resection margins (*n*, %)	1 (4.17%)	1 (4.00%)	0.999
Total PHS (days) ^*^	10 (6.8)	8 (2)	0.122
Time to Safe Discharge (days) ^*^	8.5 (5.75)	5 (1)	0.001

*IAP* intra-abdominal pressure, *EBL* estimated blood loss, *SBO* small bowel obstruction, *Total PHS* total postoperative hospital stay

*Median (InterQuartile Range)

Furthermore, LoP (min) and EBL (cc) were found to be similar between groups (*p* > 0.05), and no patients required intraoperative blood transfusion. Margin positivity rates were similar between groups (1 cT4 patient in Group 1 and 1 cT3b Group 2 patient, *p* > 0.05). IAP was raised for longer than 2–5’ (but less than 10–15’) in 4 Group 2 procedures.

Table 3 presents the 30- and 90-day postoperative complications with the use of the Clavien-Dindo system and the rates of early (≤ 30 days) readmission rates. No significant differences were found between groups. Only one early Grade III complication was recorded in Group 1 (urine leak secondary to abdominal distension, that required repositioning of the stents under radiographic guidance). Regarding 90-day complications, three patients were lost-to-follow-up. Overall, 4 Grade III complications were recorded [2 patients required intermittent self dilatations of the urostomy for stoma retraction (1 in each group) and 2 patients required nephrostomy insertion for intra- and extraluminal malignant ureteric obstruction/compression in Group 1 and 2, respectively). One Grade IV and one Grade V complication were recorded in Groups 1 and 2, respectively (IV: massive pulmonary embolism with cardiac strain—V: disseminated peritoneal and distant metastatic disease in a pT3b pN2 patient).

**Table 3:** Postoperative early (≤ 30 days) readmission and ≥ 3 Clavien-Dindo complication rates at 30 and 90 days

30 days	Group 1 – Std IAP (*n* = 24)	Group 2 – Low IAP (*n* = 25)	*P*
Total	1 (4.3%)	0 (0.0%)	0.490
Grade III	1 (4.3%)	0	
Grade IV	0	0	
Grade V	0	0	
Readmission rate	4 (17.4%)	3 (12%)	0.702

90 days	Group 1 – Std IAP (*n* = 23)	Group 2 – Low IAP (*n* = 23)	*P*
Total	3 (13.0%)	3 (13.0%)	﻿0.999
Grade III	2 (8.7%)	2 (8.7%)	
Grade IV	1 (4.3%)	0	
Grade V	0	1 (4.3%)	

*IAP* intra-abdominal pressure

Regarding postoperative pain analysis (Table 4), > 20% of the 2 × 3 table cells had < 5 count and therefore chi-square test could not be used. It was decided to merge subgroups so that 2 × 2 tables could be created to facilitate further statistical analysis (Mild/Moderate vs Severe and Mild vs Moderate/Severe). Fisher’s exact test showed higher Moderate/Severe pain levels in Group 1 over Group 2 although in general, severe pain was uncommon in both groups.

**Table 4:** Postoperative pain on Days 1 and 3

	Group 1 – Std IAP	Group 2 – Low IAP	*P*
***﻿POD1***			
Mild	1	13	
Moderate	17	10	
Severe	6	2	
**POD1**			
Mild	1 (4.2%)	13 (52.0%)	0.000
Moderate/Severe	23 (95.8%)	12 (48.0%)	
**POD1**			
Mild/Moderate	18 (75.0%)	23 (92.0%)	0.138
Severe	6 (25.0%)	2 (8.0%)	
***﻿POD3***			
Mild	9	21	
Moderate	14	4	
Severe	1	0	
**POD3**			
Mild	9 (37.5%)	21 (84.0%)	﻿0.001
Moderate/Severe	15 (62.5%)	4 (16.0%)	
**POD3**			
Mild/Moderate	23 (95.8%)	25 (100%)	﻿0.499
Severe	1 (4.2%)	0 (0%)	

*IAP* intra-abdominal pressure, *POD* post-operative day

## Discussion

The current study is, to the best of our knowledge, the first one to evaluate the role of low-pressure pneumoperitoneum in the RARC/ICUD perioperative outcomes and postoperative recovery. Our study showed that the use of low IAP led to faster bowel recovery, lower immediate postoperative pain levels and shorter time to safe discharge compared to standard pneumoperitoneum. At the same time, estimated blood loss, procedure time and rate of perioperative complications were comparable between the study groups. Although total PHS and rates of postoperative ileus and SBO were lower in the low IAP group, statistical significance was not reached.

Current literature suggests that the use of low IAP in laparoscopic procedures in general surgery such as laparoscopic cholecystectomy was not associated with higher postoperative morbidity, while at the same time the rates of postoperative shoulder pain and analgesic requirements were lower [14]. Based on these findings, the European Association for Endoscopic Surgery Guidelines recommends the use of the lowest IAP that allows adequate exposure to the operative field, rather than using a standard routine IAP [2]. Interestingly, the number of studies investigating the application of low-pressure pneumoperitoneum in robotic urological procedures is strikingly low compared to other surgical specialties [7].

A recent systematic review by West et al. published in January 2022 investigated the effect of pneumoperitoneum on clinical outcomes following urological procedures, and more specifically radical prostatectomy, live donor nephrectomy and a variety of upper tract robotic operations [7]. In their systematic review, West et al. showed that low-pressure pneumoperitoneum is safe and non-inferior to high pressure, while at the same time it can potentially reduce the levels of postoperative pain and rates of ileus.

However, it is highlighted that more research on the role of low pneumoperitoneum is needed, especially in radical cystectomy and nephrectomy. Despite technological advancements, postoperative recovery of cystectomy patients is still hindered by postoperative pain, bowel complications or sluggish bowel function. Therefore, cystectomy patients still require relatively long hospital stays, resulting in a higher risk of postoperative complications and higher procedure-related cost. Our study showed that pain levels were indeed lower when low IAP was used (admittedly, severe pain was uncommon in both groups). Ileus and small bowel obstruction rates were similar between groups, however, Low IAP patients had in general quicker bowel recovery with a significantly shorter time to first flatus and bowel movement (although statistical significance for the latter was rather marginal), and became ready for discharge more quickly.

Compared to our findings, a recent randomised controlled trial showed shorter times for first flatus and bowel movement in the robotic arm (3 and 5 days, respectively) even with standard pneumoperitoneum pressures (IAP at 12 mmHg) [15]. We feel that the larger sample size and mainly, the differences in the perioperative management of these patients can explain the differences. Certainly, it would be interesting to see whether lower pneumoperitoneum could further improve these excellent findings.

The current study showed similar operative times between the standard and low IAP groups. The effect of low IAP on total operative time remains unclear. In their systematic review, West et al. showed mixed results. Two studies on robotic prostatectomy and one study on live donor robotic nephrectomy showed longer operative times with Low IAP, a finding that was not supported by other studies, however [6, 7, 16, 17]. Although we did not record separately the operative times for the bladder removal, lymphadenectomy and reconstruction, it was felt that when difficulties occurred, they were more common during the reconstructive rather than the extirpative part of the operation, mainly due to the more limited space in the abdomen.

In the first 18 low IAP cases, pneumoperitoneum pressure would get increased to 15-18 mmHg for 2–5’, usually during the dorsal venous complex dissection in male patients. This is no longer required however, as a haemostatic suture is always applied first. Slightly longer temporary IAP increase (> 2–5’ but < 10–15’) was required in 4 Group 2 cases: 1 case of injury to the external iliac artery that required suture repair and 3 cases where limited operative field and floppy small bowel loops made the identification/isolation of the conduit loop and ileo-ileal anastomosis difficult. Only two high BMI patients required conversion from low to standard pneumoperitoneum.

In addition, our study findings can support the safety of Low-IAP use in RARC/ICUD. The frequency and severity of the intra- and postoperative complications were similar between our study groups. Finally, no difference was observed in margin positivity between the otherwise TNM stage-balanced study groups, a finding that supports the oncological safety of low-pressure cystectomy.

The main limitations of the current study need to be highlighted and include its retrospective, non-randomised and unblinded design. As such, the study is undeniably subject to selection and performance bias and its findings, although encouraging, need to be approached with caution by the readers. As a single-centre study, it reflects local experience only and has a rather small sample size. Moreover, the study findings are applicable to intracorporeal ileal conduit diversions only and as such, it is unclear whether low IAP could help with improving the postoperative outcomes of patients offered intracorporeal neobladder formation.

In addition, the possible confounding effect of the increasing surgeon’s and team’s experience should be discussed. It is known that all surgical outcomes improve with a higher number of cases and certainly this must have played a role in our findings although demographic and intraoperative parameters were comparable. Due to the small sample size, regression analysis was not performed.

Even with low IAP, hospital stay remained long at approximately a week and similar between study groups. While this is not the standard of care in various centres, the geographical limitations of the department’s catchment area that covers a total of 36000km^2^ must highlighted: it is not uncommon for otherwise fit for discharge patients to be kept in hospital until they move their bowels, their stents are removed or travel logistics are arranged. Moreover, we did not systematically assess the level of pain after POD1 and 2 (including the inability to collect data so that Morphine-Equivalent Daily Doses could be calculated), did not assess the anaesthetic/physiological impact of Low IAP and did not perform healthcare economic analysis.

Still, with its limitations, this is a study of consecutive patients operated by the same surgeon and perioperatively managed by the same team. The introduction of low IAP was shown, for the first time to the authors’ knowledge, to lead to positive patient outcomes such as the faster recovery of the bowel, lower levels of immediate postoperative pain and shorter time to safe discharge. At the same time, the new surgical approach was proven to be as safe as the previous standard of practice. We believe that our study findings definitely highlight the need for large, multi-centre, prospective, randomised and if possible, blinded, trials so that our findings could hopefully be validated and the role of low IAP in improving the postoperative recovery and outcomes of RARC patients gets clarified.

## Conclusion

The current study shows for the first time that low-pressure pneumoperitoneum RARC with intracorporeal conduit diversion can be safely offered to patients with indications for bladder removal. Patients who were operated with Low IAP had in general faster bowel recovery, lower levels of immediate postoperative pain and became stable for safe discharge more quickly. The study limitations need to be highlighted, however, and its findings should be approached with caution until a higher level of evidence is provided by larger, prospective and multicentre, randomised and blinded trials.
